# Does Right-Hemispheric Anodal tDCS Enhance the Impact of Script Training in Chronic Aphasia? A Single-Subject Experimental Study

**DOI:** 10.3389/fresc.2021.793451

**Published:** 2022-01-03

**Authors:** Mathieu Figeys, Esther Sung Kim, Tammy Hopper

**Affiliations:** ^1^Faculty of Rehabilitation Medicine, University of Alberta, Edmonton, AB, Canada; ^2^Department of Communication Sciences and Disorders, University of Alberta, Edmonton, AB, Canada

**Keywords:** aphasia, script training, transcranial direct current stimulation, right-hemispheric anodal stimulation, automaticity, tDCS, stroke rehabilitation

## Abstract

**Background:** Script training is an aphasia treatment approach that has been demonstrated to have a positive effect on communication of individuals with aphasia; however, it is time intensive as a therapeutic modality. To augment therapy-induced neuroplasticity, transcranial direct current stimulation (tDCS) may be implemented. tDCS has been paired with other speech-language treatments, however, has not been investigated with script training.

**Aims:** The purpose of this study was to determine if tDCS improves communication proficiency when paired with script training, compared to script training alone.

**Methods and Procedures:** A single-subject experimental design was implemented with a participant with non-fluent aphasia, using two scripts across treatment conditions: script training with sham-tDCS, and script training with anodal-tDCS. Treatment sessions were 75 min long, administered three times weekly. Anodal tDCS was implemented for 20 min with a current of 1.5 mA over the right inferior frontal gyrus.

**Results:** Large effect sizes were obtained on script mastery for both stimulation conditions (anodal *d*_2_ = 9.94; sham *d*_2_ = 11.93). tDCS did not improve script accuracy, however, there was a significant improvement in the rate of change of script pace relative to baseline (3.99 seconds/day, *p* < 0.001) in the anodal tDCS condition.

**Conclusion:** Despite a null tDCS result on accuracy, the script training protocol increased script performance to a near-fluent level of communication. There is preliminary evidence to suggest that tDCS may alter the rate of script acquisition, however, further research to corroborate this finding is required. Implications for future studies are discussed.

## Introduction

Aphasia is an acquired language impairment primarily caused by cerebrovascular accidents involving the left middle cerebral artery. Aphasia can cause significant communication deficits; even mild aphasia can have significant deleterious effects on a person's ability to participate in everyday life activities and fulfill social roles (1). Approximately one-third of people who survive a cerebrovascular accident will experience some degree of aphasia (2). To date, there is no cure for aphasia, however, several behavioral treatments exist that help improve language and communication specific to aphasia (2). One functional speech-language rehabilitative approach is script training, which has emerged as a potentially promising treatment option for aphasia.

Script training in aphasia typically involves the repeated practice of words, phrases, and sentences embedded within a monolog or dialogue that is individualized to the person with aphasia (3). People with aphasia (PWA) engage in repeated script practice using a fading of cues protocol until they can speak the script automatically and use it in everyday communication situations (4, 5). Script training is based on the Instance Theory of Automatization (6), which posits that automaticity of skills is achieved by retrieving intact, context-dependent information (in this case, scripts of language) from long-term memory. Scripts are encoded through repetition, and typically the clinician uses a fading cues protocol until the PWA can recall and speak the script automatically (5, 6). Since the development of script training protocols, there has been an increasing literature base exploring the effectiveness of script training in PWA. Most have been single-subject experimental designs or case series of individuals with chronic aphasia of mild-moderate severity (5, 7). However, studies employing larger samples also demonstrate positive treatment outcomes related to spoken and written language and communication (8–10).

One caveat for the use of script training is that it requires extensive practice and time. For example, 22–44 in-person formal training sessions have been reported to master three scripts, excluding individual time practicing at home (4, 5). Some attempts have been made to use technology to increase the efficiency of training [i.e., virtual therapist (8)], but PWA may still need to devote significant time and cognitive effort in the learning process. One potential method to assist in increasing efficiency of the script training approach is through neuromodulation using transcranial direct current stimulation (tDCS).

### tDCS and Aphasia Treatment

Transcranial direct current stimulation (tDCS) involves the application of a low-dose electrical current across the brain to alter neuronal transmembrane polarities (11). This subthreshold current is not strong enough to invoke an action potential, however, it is proposed to hypopolarize or hyperpolarize the neuronal resting state to achieve heightened sensitivity or dampening in cortical regions of interest (11).

tDCS applications should include the consideration of factors such as the selection of an appropriate region of stimulation, current strength, duration, and frequency. Another consideration specific to stroke-acquired aphasia is the size and location of the lesion, as the distribution of the current can be altered by this lesioned tissue (12). Most tDCS studies for aphasia have utilized anodal stimulation over the perilesional structures in the left-hemisphere, and/or cathodal stimulation over the right-hemisphere (13).

In addition to targeting perilesional tissue with anodal tDCS, there is increasing evidence to suggest other electrode montages are beneficial for PWA. For instance, left-cathodal, as well as right-anodal stimulation may promote secondary language processing within the right-hemisphere (14–21). Anodal-tDCS over the right-hemisphere paired with speech-language treatment has been utilized in previous aphasia studies with reports of increased verbal fluency (17) and naming ability (18). When left-hemisphere damage is extensive, the right-hemisphere may become the dominant language promoter (22). Therefore, right-hemispheric anodal-stimulation may increase language performance if the right-hemisphere has become the primary neural-area for language processing [i.e., after a large left-hemisphere lesion (23)].

Indeed, previous studies have demonstrated that tDCS stimulation over the right inferior frontal gyrus can improve language functioning (17, 18). The right inferior frontal gyrus is suggested to be involved in homologous speech-language processing, such as singing and intonation, by incorporating the right arcuate fasciculus and communicating structures; these neural pathways are implicated in melodic intonation therapy for aphasia (17, 24, 25). By inducing tDCS-neuromodulation to these areas, language abilities may be improved, particularly when paired with speech-language therapies (including script training), to enhance and/or expedite treatment effects.

Script training has been demonstrated to be an effective treatment option in improving functional communication in PWA. Whereas, tDCS may improve the efficiency and efficacy of script training, it has not been examined together with script training. Therefore, the purpose of this study was to determine if outcomes from script training could be enhanced when combined with tDCS in an individual with post-stroke aphasia. An anodal right-hemispheric montage was selected, as the presence of a large left-hemisphere lesion precluded left-hemisphere placement. The specific research questions were as follows:

1. *What are the effects of script training for an individual with post-stroke aphasia with an extensive left-hemispheric lesion?*
2. *What are the effects of combining anodal right-hemispheric tDCS over the inferior frontal gyrus with script training for an individual with chronic post-stroke aphasia with an extensive left-hemispheric lesion?*

## Materials and Methods

### Design

A *n*-of-1, single-blinded A - B - B+C - A design was implemented where “A” indicates conventional baseline measures, “B” indicates script training paired with sham-tDCS, and “B+C” represents script training with active anodal-tDCS.

### Participant

**Figure 1:**
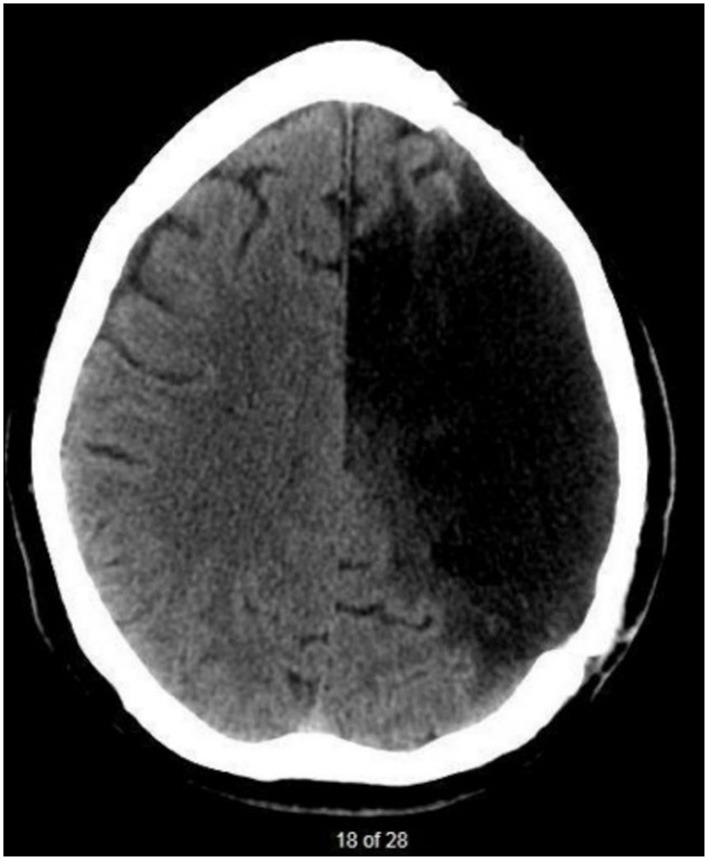
CT scan taken 3 years after JB's stroke. There is evidence of a large, chronic post-stroke lesion within the left-hemisphere. Regions affected are associated with branches of the left middle cerebral artery.

The participant (JB) was recruited based on convenience sampling. JB, was a 45-year-old English-speaking male, 3 years, 11 months post-onset of a large left-hemispheric stroke resulting in chronic aphasia, apraxia of speech and right-sided hemiplegia. His lesion encompassed the left parietal, temporal, and frontal lobes seen on neuroimaging (Figure 1). JB had 4 years of post-secondary education and worked as a project manager prior to the stroke.

Prior to commencing the study, JB completed screenings of vision: (Rosenbaum pocket vision screener; 20/20 bilaterally uncorrected at 14″ distance), hearing [minimal-pairs discrimination task using words/non-words adapted from *PALPA* 1 and 2 (26); 98% correct] and non-verbal fluid intelligence [Raven's Coloured Progressive Matrices (27); 34/37 correct]. The severity and profile of aphasia were characterized by the *WAB-Revised* [WAB-R (28)]. JB presented with non-fluent (Broca's) aphasia with a WAB-R Aphasia Quotient of 51.7. Auditory comprehension was a relative strength. Verbal expression consisted of mainly single words spoken in a slow, halting manner. JB used frequent gestures as well as a tablet-based augmentative and alternative communication program with pictographic and synthesized voice support to supplement his limited spoken language.

### Ethics

This project was approved by the University of Alberta Research Ethics Board (Pro00054921). Study procedures were explained to JB and his spouse in written and verbal form using supported communication strategies, and JB provided signed informed consent. The participant received a fifty-dollar gift card as compensation for participating in the study.

### Intervention

#### Script Training

In collaboration with JB, two dialogue-based scripts were developed using standardized templates adapted from Kaye and Cherney (3). As our participant had a limited timeframe to participate in this study, six participant-phrases per script were selected from the standardized templates and personalized. After script personalization, Script 1 had a Flesch-Kincaid reading level of 1.4, and Script 2 had a reading level of 1.2.

Five baseline probes (Phase A) were administered for Script 1 prior to initiating treatment. During Phase B, while Script 1 was being trained, probes were conducted for Script 2. During each probe, the participant had access to the printed script and attempted to read it aloud. No training or feedback was given during probes.

During the treatment phases (B, B+C), a fading cue-hierarchy protocol (4) was used during the script training. Scripts were taught one phrase at a time, using a dialogue-turn format (Table 1). Throughout the script training procedure, JB had access to the whole printed script in addition to cue cards with one script line written on each. Independent spoken production of the script was defined as the participant correctly saying the phrase aloud, without cueing. The participant was required to independently say the phrase aloud 20 times before the next phase in the script was added to the previously mastered phrase(s). The researcher immediately corrected errors (defined as any distortion of a word or deviation in syntactic structure), and the individual repeated the word or phrase aloud. Two scripts were taught sequentially; JB chose the order. Script 1 (ordering a pizza) was first during treatment phase B; Script 2 (grocery shopping) was second in phase B+C. A minimum of three sessions in each phase was required, however, no limit on treatment sessions was set for either script. Rather, a script was considered mastered after JB demonstrated two consecutive sessions with over 90% percent of script correct (4, 5).

**Table 1:** Script Training Procedures Implemented.

**Step**	**Description**
1	Phrase modeling by the researcher
2	Reading of the phrase between the client and researcher in unison
3	Reading of the phrase in unison, with the researcher slowly fading their voice out
4	Independent phrase production by the client (with cueing)
5	Independent phrase production by the client (without cueing)
6	After 20 successful independent productions of the phrase, the next phrase is added on to the mastered phrases

*[Adapted from Youmans et al. (4)]*.

During B and B+C, probing continued at the beginning of each session prior to script training. When mastery was achieved for Script 1, Script 2 training was initiated. Training on Script 1 was discontinued, although the participant continued to practice Script 1 at home. After demonstrating mastery of Script 2, script training was discontinued. Four maintenance data points were collected at one, five, seven, and fourteen weeks after completing treatment phase B+C. Maintenance data probes were conducted the same way as baseline data probes, with the participant having the printed script and attempting to read it aloud. No feedback or cueing was provided.

The protocol was conducted in person three times per week, in 75-min sessions at a university research lab. Homework was assigned in the form of 15-min of script practice per day. Audio files using the fading-of-cue protocol were recorded for both scripts on JB's tablet at the start of each training phase to facilitate script training at home. The participant kept a homework log to record daily practice.

#### tDCS Stimulation

In the anodal-tDCS condition, a 1.5 mA current for 20-min was applied through 5 cm × 7 cm electrode sponges, saturated in 10 mL of 0.9% NaCl solution, using a Magstim HDCStim device. The anodal-electrode was placed over the right inferior frontal gyrus, determined to be at the intersection of T4-Fz and F8-Cz (29), and secured with a hairnet. The cathodal electrode was placed on the left deltoid muscle (30).

For a blinding procedure during phase B, a 1.5 mA current was applied for 1 min with a 15-second ramp-up and ramp-down period, to create the sensation of electrical stimulation (31). tDCS was implemented for the first 20 min of the script training period, and the electrodes were taken off at the end of the session.

### Dependent Measures

Three outcome measures were used to examine treatment effects: (1) script mastery (defined as 90% of script spoken correctly over two consecutive sessions); (2) total time to complete script turns; and (3) the number of sessions required to demonstrate script mastery.

Script mastery was measured using both binary scoring (Correct/Incorrect) and the Naming and Oral Reading for Language in Aphasia 6-Point Scale [NORLA-6 (32)]. Using this scale, each word in the script is scored from 0 to 5, where 0 indicates no communicative output, and 5 indicates a perfect response. NORLA-6 scoring provided a more fine-grained analysis of script production, accounting for speech/language errors (such as delays in production, distortions of words, tenses, morphemes, and phonemes). Total time to complete script turns was calculated based on the sum of time JB required to complete his turns in the scripted dialogue. A turn was defined by the end of the researcher's probe until the end of JB's turn.

### Analyses

In line with single-subject research designs, traditional visual analysis methods [outlined in Kratochwill et al. (33)] and effect sizes [using the modified Cohen's *d*_2_ as described in Beeson and Robey (34)] using baseline and post-treatment scores for each script are reported. The magnitude of effect sizes will be interpreted as 2.6, 3.9, and 5.8 for small, moderate, and large effect sizes respectively (34, 35). In addition, a modeling technique to account for change across time—an Interrupted Time Series Analysis [ITSA (36)] was also used.

ITSA can determine if significant differences exist between the slopes (trends) and levels (corresponding y-values) in time-series designs. ITSA is advantageous as it can be utilized for between group comparisons, and with multiple treatment phases across time (36). ITSA accounts for autocorrelation and utilizes an ordinary least squares regression process to create a regression-based model of the dataset while calculating pre- and post-intervention levels and trends after an intervention is started or withdrawn. An ITSA package (37) available for Stata (38) from the Statistical Software Archive was used to run the ITSA models.

## Results

### Interrater Reliability

A second coder independently scored each word (using binary and NORLA-6 scoring) for both scripts across conditions, for nine sessions chosen at random. The individual was trained on the NORLA-6 scale by the primary author. A Krippendorff's alpha was calculated in SPSS (IBM Corp., Version 24, Armonk, NY). An α = 0.99 was obtained, suggesting strong interrater reliability between both raters across scoring methods.

### Script Mastery

#### Binary Scoring

**Figure 2:**
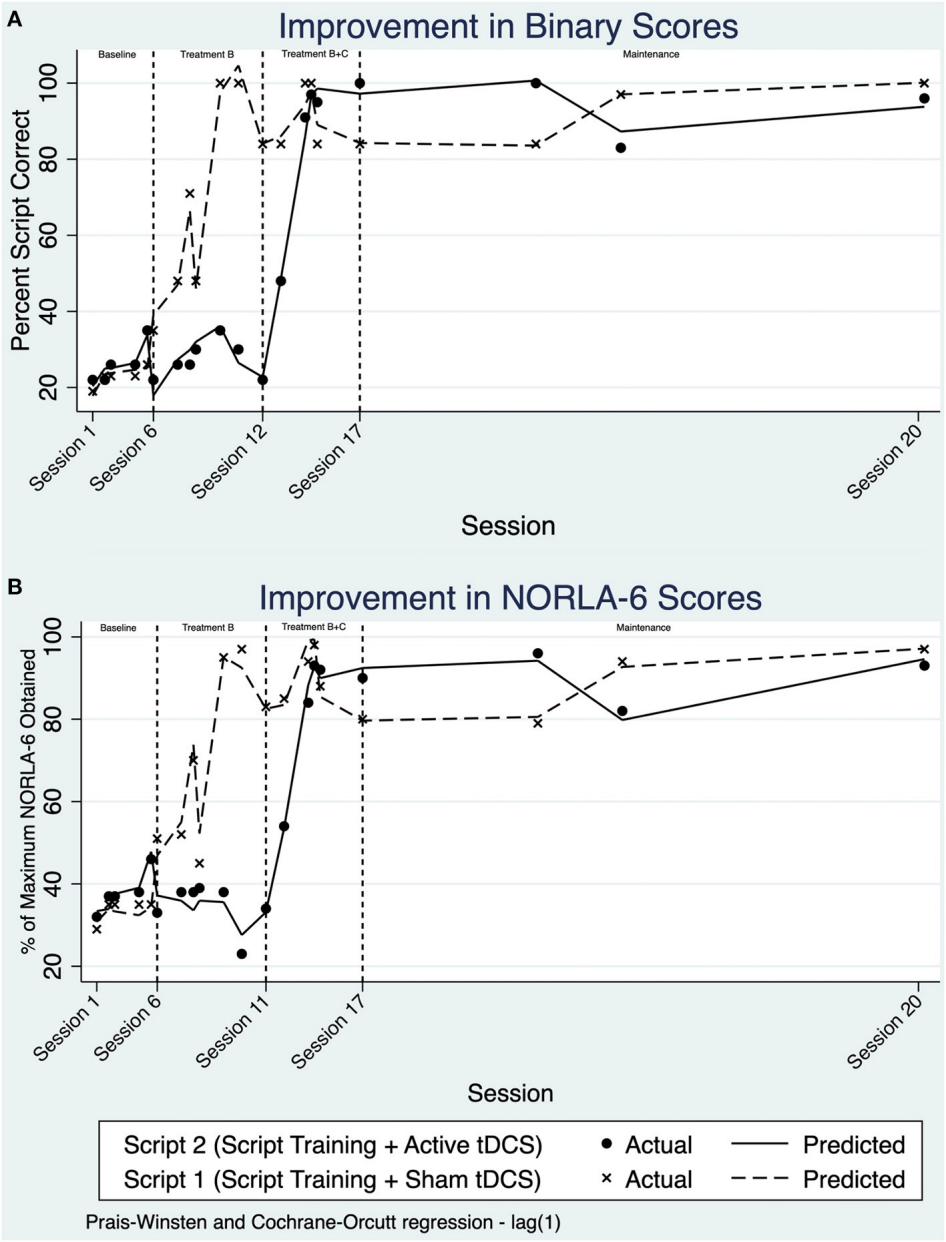
**(A)** Line chart of percent script correct using binary scoring over treatment sessions and maintenance. **(B)** Line chart of percent script correct using NORLA-6 scoring over treatment sessions and maintenance.

Scores were transformed to percent script correct based on the sum of words correct and the total number of words in the script. Upon visual analysis, a stable pattern is noted during the initial baseline period across both study conditions (Figure 2A). Using ITSA, a non-significant difference between both scripts during the baseline period was calculated, indicating similar performance between both scripts during the baseline period.

Applying ITSA across study phase B, there was an increase in the slope and change in level for Script 1. This change in daily trend was determined to be 1.54%/day (*p* = 0.034, 95% CI [0.12, 2.96]). That is, JB was improving at a rate of 1.54% /day compared to baseline on the trained script. Script 2, which remained untrained, remained comparable to the baseline period (−0.40%/day; *p* = 0.685, 95% CI [−2.44, 1.63]). Applying ITSA across phase B+C, there appears to be an increase in performance for Script 2. Indeed, the change in daily trend within treatment phase B+C for Script 2 was significant, increasing 1.71%/day relative to baseline (*p* = 0.021, 95% CI [0.39, 3.02]). When comparing the slopes before and after anodal tDCS was administered, no significant differences in the interaction of group and intervention phase was observed.

A post-trend analysis within ITSA using a linear combination of estimators (*lincom*) revealed no significant difference between script training with anodal-tDCS compared to script training with sham-tDCS in the maintenance period. This suggests no significant difference between retention of the scripts across sham and anodal conditions during the maintenance phase.

Large effect sizes were seen for both treatment conditions; the effect size for script training with sham-tDCS was *d*_2_ = 9.94, compared to script training with anodal-tDCS *d*_2_ = 11.93. Despite the large effect sizes, there remained a lack of statistical significance from ITSA when comparing between both conditions (slopes of 1.54%/day for Script 1, compared to 1.71%/day for Script 2).

#### NORLA-6 Scoring

NORLA-6 scores were transformed to a percentage, based on the maximum NORLA-6 score achievable. Visual analysis of the NORLA-6 model complements the results of the binary scoring method. In the baseline period, both scripts appear to be stable without any significant fluctuations in performance (Figure 2B). Within treatment phase B, NORLA-6 scores for Script 1 improved significantly, evident by the rise in trend, and change in level. Script 2 mastery continued to demonstrate a baseline-like pattern, without any significant changes, until the initiation of B+C. Script 2 also had a rise in trend and overall level in phase B+C, with script 1 remaining relatively stable. This stability was further extended into the maintenance period.

With ITSA, there were no significant differences between treatment conditions with NORLA-6 scoring. Further, no significant differences in the interaction of group and intervention phase were observed. Within the maintenance period, there was no significant difference between the anodal-tDCS and sham-tDCS phases using *lincom* post-trend analysis. Like binary scoring, a large effect size was obtained for script training with sham-tDCS (*d*_2_ = 8.88), and a moderate effect size was seen in script training paired with anodal-tDCS (*d*_2_ = 12.23). tDCS did not impact script retention, evidenced by similar performance across scripts during the maintenance period.

### Time to Complete Scripts

**Figure 3:**
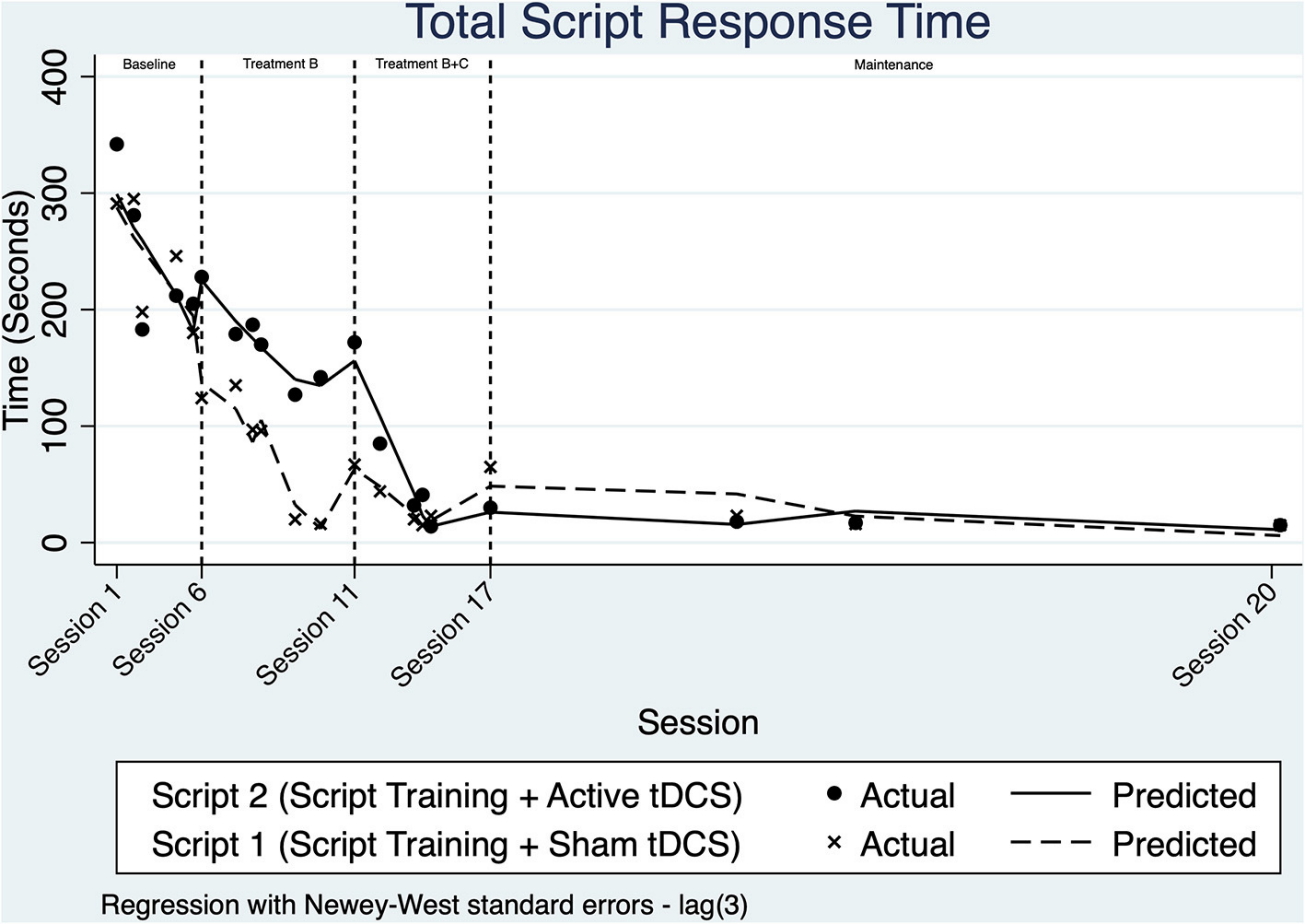
Line chart of the change in total time (in seconds) required to complete script phrases.

Visual analysis of Figure 3 suggests that the time needed to complete script turns decreased across both scripts starting within the baseline phase, suggesting a potential learning effect prior to implementing script training.

Once intervention phase B was implemented, the trend for script 1 decreased at a faster rate than seen during baseline. Script 2 also appears to become faster over this phase. The daily rate of change across phase B for Script 1 was modeled with ITSA to improve by an additional 3.68 seconds/day relative to the baseline (*p* = 0.303, 95% CI [−3.56, 10.93]).

Upon visual analysis across treatment phase B+C, there is a change in level after a marked decrease in trend for script 2. A non-significant change in daily trend and level is evident within treatment B+C for script 1. ITSA modeled the change in slope for Script 2 time relative to baseline to be 3.99 s/day (*p* < 0.001, 95% CI [2.44, 5.56]). When examining the interaction of group and intervention phase (non-relative to baseline), no significant differences were noted.

Time to complete script turns remained stable during the maintenance period for both scripts. ITSA modeling revealed that there were no statistical differences between the time taken to speak the two scripts during the maintenance period. Effect size measures for both conditions were similar, with a *d*_2_ = −5.31 for script training with sham-tDCS, and a *d*_2_ = −3.42 for script training paired with anodal-tDCS.

### Number of Sessions to Achieve Mastery

JB achieved script mastery, defined as speaking 90% of the script correctly and independently over two consecutive sessions, after six sessions for Script 1 (paired with sham-tDCS) and five sessions for Script 2 (paired with anodal-tDCS).

## Discussion

The study participant demonstrated positive outcomes related to script mastery, total time to complete script turns, and the number of sessions to mastery in both treatment conditions in this study. There was no difference in script mastery or total script time between the two treatment conditions. There is preliminary evidence from the ITSA analysis that the use of tDCS may have facilitated faster learning of the second script, however, the generalizability of this result is limited due to the single-subject design. The findings of this study are important from both a methodological perspective and may inform future research designs.

The results of this study are consistent with the existing literature regarding the variable effects of tDCS in aphasia protocols [see (39–41)]. These conflicting results may be explained by differences in the tDCS treatment parameters used across studies, heterogeneity among participants, variable treatment paradigms, and methodological differences (39). Several factors may have contributed to the obtained results of tDCS paired with script training. First, tDCS placement may have mitigated the potential benefits of neuromodulation. Anodal-tDCS over the right IFG was selected based on the model proposed by Anglade et al. (23). In the absence of neuroimaging-guided tDCS placement (42), it is unknown whether the right IFG was the optimal stimulation site for our participant. Stimulation in more posterior brain structures, such as the superior temporal gyrus, could potentially engage a greater homologous related ventral stream response [refer to Hickok and Poeppel (43) for a discussion on dorsal and ventral streams]. Further research is necessary to determine optimal montage, target structures of interest, as well as hemispheric effects and the individualization of tDCS protocols.

Second, the design of the study may have resulted in sub-optimal effects of the tDCS. In the current study, sessions were not consecutive. Thus, the effects of tDCS may have diminished between sessions. In previous studies, the frequency of tDCS stimulation, as well as stimulation parameters have varied (12, 22), and it remains unclear if the frequency of tDCS stimulation is correlated to an improvement in learning and language performance. For instance, Monti et al. (14) report increased naming accuracy in individuals after a single session of left-hemispheric cathodal stimulation (2 mA for 10 min). In contrast, Spielmann et al. (40) reported no significant changes in individuals who received left-hemisphere anodal tDCS for 5 consecutively administered sessions per week, over 2 weeks (1 mA for 20 min).

Third, floor and ceiling effects are present in the dataset; once JB mastered the scripts, he consistently achieved near-perfect performance. Although the scripts were challenging at first, they may have been too easy such that any potential differential effect of the use of tDCS on learning accuracy was masked. We noted that time to complete both scripts decreased during the baseline probes and treatment phase B, suggesting learning/practice effects for the untreated script. Upon closer review, there were no corresponding improvements in accuracy for the untreated script, and the reduced time reflected reductions in speech breaks/pauses. ITSA revealed only the trained scripts showed significant changes relative to baseline (Figure 2) during the treatment phases. Thus, it seems explicit training of the scripts was necessary to increase script accuracy, however, mass exposure without training may reduce script time due to potential learning and habituation effects.

Other design considerations may impact future study results, for example, adding additional treatment phases and washout periods (44). Future studies should examine the effects of an untrained script and include other speech-language tasks. Due to participant time constraints, it was decided to provide sham tDCS first to remove the need for a washout period. Thus, the research team was not blinded. Double-blinding is recommended in future studies. Further research pairing script training with the use of neuroimaging-based approaches is recommended to examine larger neuronal networks utilized in script training acquisition and in long-term maintenance. In addition, structural imaging can be utilized to model tDCS electrical fields on each participant to optimize electrode placement. Furthermore, brain-derived neurotrophic factor genotyping would be of benefit to optimize tDCS for PWA (45). Finally, other factors including neuropharmacological agents were not controlled for and may have physiologically impacted the effects of tDCS (46).

Despite the null tDCS results on script accuracy, our participant demonstrated significant gains on the two practiced scripts due to the script training protocol. These successful script training results are consistent with previously reported studies of patients with similar aphasia profiles (4, 7) and add to the existing literature in this area. Further, we extend the literature on the topic of the personalization of scripts. Personalization of scripts may increase communicative gains to a greater extent than non-personalized scripts (47). In this study, scripts were personalized regarding names, places, and favorite foods in JB's environment. Participant reports and detailed logs from our participant demonstrated significant interest in both scripts. JB was very motivated and continues to individually practice the scripts after completion of the study. This demonstrates an individualized functional benefit of the implemented script training protocol for our participant.

In conclusion, script training had a positive effect on communication for an individual with post-stroke aphasia with a large left-hemispheric lesion. The addition of tDCS did not increase functional communication, including the time required to complete the script or script accuracy. Script training, as well as tDCS applications, may be a promising rehabilitation approach to assist in communicative compensation for individuals with post-stroke aphasia, however, due to limitations in this study, future research is necessary, with a focus on larger sample sizes.

## Data Availability Statement

The original contributions presented in the study are included in the article/Supplementary Materials, further inquiries can be directed to the corresponding author.

## Ethics Statement

The studies involving human participants were reviewed and approved by University of Alberta. The patients/participants provided their written informed consent to participate in this study. Written informed consent was obtained from the individual(s) for the publication of any potentially identifiable images or data included in this article.

## Author Contributions

MF, EK, and TH contributed to the conception, design of the study, and wrote sections of the manuscript. MF was involved in data collection, intervention administration, and data analysis. EK and TH provided guidance and supervision. All authors contributed to manuscript revision, read, and approved the submitted version.

## Conflict of Interest

The authors declare that the research was conducted in the absence of any commercial or financial relationships that could be construed as a potential conflict of interest.

## Publisher's Note

All claims expressed in this article are solely those of the authors and do not necessarily represent those of their affiliated organizations, or those of the publisher, the editors and the reviewers. Any product that may be evaluated in this article, or claim that may be made by its manufacturer, is not guaranteed or endorsed by the publisher.
