# Supplementary material for: Does Right-Hemispheric Anodal tDCS Enhance the Impact of Script Training in Chronic Aphasia? A Single-Subject Experimental Study
Source: Front Rehabil Sci. 2022 Jan 3;2:793451. doi: 10.3389/fresc.2021.793451 (PMC9397953; doi:10.3389/fresc.2021.793451)
Supplement: Supplementary file 1 [file Data_Sheet_1.DOCX]

**Supplementary Material**

**Appendix A: Scripts Adapted from Kaye & Cherney, 2016** (3)

**Script 1 (Level 1): Restaurant/Pizza**

**Researcher: Welcome. Is this your first time here?**

No*.* We want to order a pizza.

**Researcher: What kind of pizza would you like?**

Royal Special, No olives.

**Researcher: What Size?**

What’s enough for two?

**Researcher: A large pizza is plenty.**

That sounds about the right size.

**Researcher: Anything else?**

Can we get the check?

**Researcher: How did you like our pizza?**

The best in (*CITY*).

**Script 2 (Level 1): Grocery Shopping**

**Researcher: Want to go grocery shopping?**

Sure, (*NAME*). And let’s go soon.

**Researcher: What’s in the fridge now?**

Leftover pasta.

**Researcher: When should we go?**

How about six o’clock?

**Researcher: Don’t we need some milk?**

I think you’re right.

**Researcher: I can cook.**

Then I’ll clean up.

**Researcher: How about a walk afterwards?**

That sounds perfect.
