# Supplementary material for: Does Right-Hemispheric Anodal tDCS Enhance the Impact of Script Training in Chronic Aphasia? A Single-Subject Experimental Study
Source: Front Rehabil Sci. 2022 Jan 3;2:793451. doi: 10.3389/fresc.2021.793451 (PMC9397953; doi:10.3389/fresc.2021.793451)
Supplement: Supplementary file 2 [file Data_Sheet_2.DOCX]

**Script 1: Raw Data**

| Session | Phase | NORLA-6 Score (%) | Binary Score (%) | Time (s) |
| --- | --- | --- | --- | --- |
| 1 | A1 | 29.03 | 19.35 | 333 |
| 2 | A2 | 34.84 | 22.58 | 295 |
| 3 | A3 | 34.84 | 19.35 | 198 |
| 4 | A4 | 35.48 | 22.58 | 246 |
| 5 | A5 | 35.48 | 22.58 | 180 |
| 6 | B1 | 40.64 | 35.48 | 124 |
| 7 | B2 | 51.61 | 48.39 | 135 |
| 8 | B3 | 54.19 | 48.39 | 97 |
| 9 | B4 | 69.68 | 64.52 | 96 |
| 10 | B5 | 95.48 | 100 | 20 |
| 11 | B6 | 98.71 | 100 | 16 |
| 12 | B+C1 | 84.52 | 83.87 | 69 |
| 13 | B+C2 | 85.16 | 83.87 | 44 |
| 14 | B+C3 | 94.19 | 100 | 20 |
| 15 | B+C4 | 98.06 | 100 | 15 |
| 16 | B+C5 | 84.52 | 83.87 | 23 |
| 17 | M1 | 80 | 83.87 | 65 |
| 18 | M2 | 79.35 | 83.87 | 23 |
| 19 | M3 | 94.19 | 100 | 16 |
| 20 | M4 | 96.78 | 100 | 17 |

**A:** Baseline Measures (without the treatment intervention being provided), **B (in red text):** Implementation of script training on Script 1 paired with sham tDCS. Script 2 remained untrained. **B+C:** Implementation of script training on script 2 paired with anodal tDCS. Training on script 1 stopped when this phase began. **M:** Maintenance period. No treatment interventions or training was provided. Rows shaded in blue were utilized to calculate Cohen’s d_2_ effect sizes outlined in (34).

**Script 2: Raw Data**

| Session | Phase | NORLA-6 SCORE (%) | BINARY SCORE (%) | TIME (S) |
| --- | --- | --- | --- | --- |
| 1 | A1 | 32.17 | 21.74 | 342 |
| 2 | A2 | 36.52 | 21.74 | 281 |
| 3 | A3 | 35.65 | 26.09 | 183 |
| 4 | A4 | 38.26 | 26.09 | 212 |
| 5 | A5 | 46.09 | 34.78 | 205 |
| 6 | B1 | 33.04 | 21.74 | 228 |
| 7 | B2 | 37.39 | 26.08 | 179 |
| 8 | B3 | 38.36 | 26.08 | 187 |
| 9 | B4 | 39.13 | 30.44 | 170 |
| 10 | B5 | 38.26 | 34.78 | 127 |
| 11 | B6 | 38.26 | 30.43 | 142 |
| 12 | B+C1 | 33.91 | 21.74 | 148 |
| 13 | B+C2 | 53.91 | 47.83 | 85 |
| 14 | B+C3 | 84.35 | 91.3 | 32 |
| 15 | B+C4 | 93.04 | 95.65 | 41 |
| 16 | B+C5 | 92.17 | 95.65 | 14 |
| 17 | M1 | 90.43 | 95.65 | 30 |
| 18 | M2 | 95.65 | 100 | 18 |
| 19 | M3 | 81.74 | 82.61 | 17 |
| 20 | M4 | 93.04 | 95.65 | 15 |

**A:** Baseline Measures (without the treatment intervention being provided), **B:** Implementation of script training on Script 1 paired with sham tDCS. Script 2 remained untrained. **B+C (in red text):** Implementation of script training on script 2 paired with anodal tDCS. Training on script 1 stopped when this phase began. **M:** Maintenance period. No treatment interventions or training was provided. Rows shaded in blue were utilized to calculate Cohen’s d_2_ effect sizes outlined in (34).
